# Optimizing CABG in Brazil: Why Superspecialization and Personalized Care Matter Now

**DOI:** 10.21470/1678-9741-2026-0183

**Published:** 2026-06-12

**Authors:** Omar Asdrúbal Vilca Mejia, Rodrigo Coelho Segalote, Maurilio Onofre Deininger, Bruno Mahler Mioto, Walter José Gomes

**Affiliations:** 1 Cardiovascular Surgery Department, Instituto do Coração, Hospital das Clínicas, Faculdade de Medicina, Universidade de São Paulo, São Paulo, São Paulo, Brazil; 2 Instituto Nacional de Cardiologia, Rio de Janeiro, Rio de Janeiro, Brazil; 3 Hospital Alberto Urquiza Wanderley, João Pessoa, Paraíba, Brazil; 4 Instituto do Coração, Hospital das Clínicas, Faculdade de Medicina, Universidade de São Paulo, São Paulo, São Paulo, Brazil; 5 Surgery Department, Universidade Federal de São Paulo, São Paulo, São Paulo, Brazil

**Figure:**
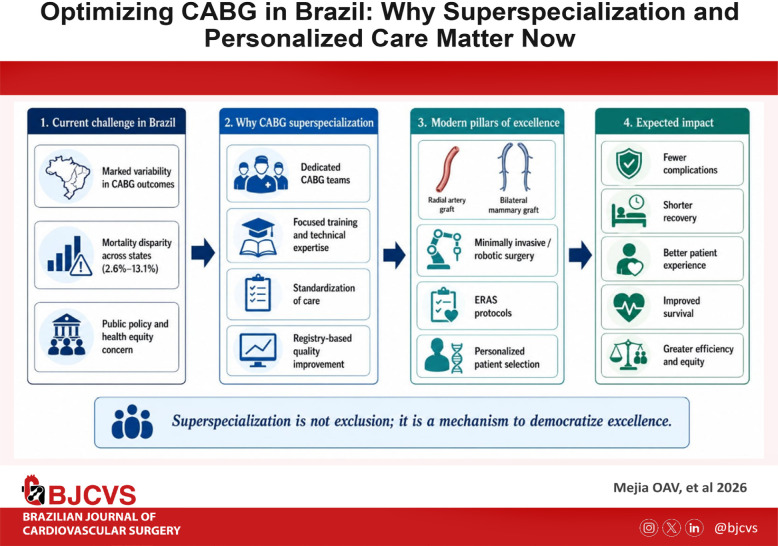

Coronary artery bypass grafting (CABG) remains the gold standard for complex coronary artery disease (CAD). However, significant variability in outcomes in Brazil, evidenced by the Departamento de Informação e Informática do Sistema Único de Saúde (DATASUS) and the BYPASS/REPLICCAR registries, demands the importance of focused training and dedicated expertise in achieving optimal results in CABG. This editorial argues for the formal recognition of CABG as a superspeciality - not as a means of professional exclusion, but as a path to democratize excellence, by integrating minimally invasive techniques, and the ERAS protocol, and by anchoring these advancements in multicenter evidence. In Brazil, between 2008 and 2017, death rates by state ranged from 2.6% to 13.1% for isolated CABG^[1]^. This disparity is no longer just a clinical challenge; it is a public policy crisis that demands the formal recognition of CABG as a dedicated superspeciality within cardiovascular surgery. In modern medicine, superspecialization is indispensable, and in cardiac surgery this is no different, in the pursuit of better results within personalized medicine and the sustainability of healthcare systems where state of the art brings quality, safety, and efficiency. Programs dedicated to training in CABG can lead to increased expertise, faster operations, greater use of arterial grafts, fewer complications, improved patient experience, and better survival rates^[2-4]^.

## The Burden of Coronary Artery Disease in Brazil

CAD is the primary cause of death in Brazil, causing roughly 171,000 to 400,000 deaths annually. The number of individuals with ischemic heart disease increased from 1.48 million in 1990 to over 4 million in 2019, driven by population aging^[5]^. Within this landscape, CABG is not merely a surgical procedure, it is a vital public health intervention. Despite the rise of percutaneous coronary interventions, CABG continues to offer superior long-term survival for patients with complex disease, ischemic myocardial dysfunction, diabetes, and left main involvement^[6]^.

However, the delivery of this *gold standard* is marred by deep-seated inequalities. To move from a model of *routine cardiac surgery* to one of *specialized precision*, Brazil must adopt a policy of superspecialization. This movement is a collective invitation for the cardiovascular surgical community to elevate the procedure from a technical act to a comprehensive journey of care, ensuring that the *gold standard* is a reality for all, regardless of geographic location.

## Dissecting the Evidence: The Reality of Brazilian Registries

CAD remains the leading cause of death globally and a massive burden on the Brazilian Unified Health System, the Sistema Único de Saúde (SUS). While CABG is an established pillar of treatment, current data from DATASUS reveal a sobering reality: significant regional heterogeneity in surgical outcomes. In a country of continental dimensions, mortality rates for the same procedure vary from 2.6% to over 13.1% depending on the state and the hospital^[1]^. This disparity is no longer just a clinical challenge, it is a public policy problem that demands the formal recognition of CABG as a dedicated superspeciality within cardiovascular surgery.

Furthermore, if we compare elective isolated myocardial revascularization surgeries from the last 10 years (2015 - 2025), we can see that there was a recovery in volume (10,406 in 2015 and 11,059 in 2025), but there was no change in the median length of hospital stay (9.9 days in 2015 and 9.4 days in 2025) nor in unadjusted hospital mortality (5.3% in 2015 and 5.4% in 2025)^[7]^. During the same period, the average reimbursement amount from the SUS per patient more than doubled (R$11.644,35 in 2015 to R$25.968,26 in 2025). When comparing these data with results from American and European registries, we observe that, although the risk profile has become more severe over time, they have managed to reduce hospital stay times and hospital mortality rates^[8,9]^. The lack of training programs in the area, as well as the implementation of metrics related to the state of the art in CABG, the incorporation of evidence-based perioperative optimization protocols, and the lack of implementation of patient-reported outcomes and experiences may be hindering the continuous quality improvement of CABG's results at the national level^[10]^.

The *BYPASS Registry*, an initiative by the Sociedade Brasileira de Cirurgia Cardiovascular (or SBCCV)^[11]^, confirmed that the Brazilian CABG patient is increasingly complex - often presenting with diabetes, prior myocardial infarction, and advanced age. The data shows that when surgery is performed by dedicated teams using arterial grafts, results improve significantly. However, few of the patients underwent off-pump CABG (OPCAB) (13%). Bilateral internal mammary artery (BIMA) was used only in 4.8%, and the radial artery in 1.1%. Unadjusted operative mortality was 2.8%, and the incidence of cerebrovascular accident was 1.2%. In addition to the data not being adjusted, information on the efficiency of care was not reported, such as extubation time, intensive care unit (ICU) length of stay, and hospital stay. A low percentage of use of two or more arterial grafts was reported, despite the known impact on patient survival. On the other hand, mortality data from the BYPASS registry cannot be compared with those from DATASUS because it is a voluntary registry.

The *REPLICCAR project* (the Cardiovascular Surgery Registry of the state of São Paulo), funded by Fundação de Amparo à Pesquisa do Estado de São Paulo (or FAPESP) and in partnership with Harvard University, aimed to improve outcomes in CABG surgeries^[12]^. The project demonstrated that, through a set of quality measures, there was a significant reduction in intubation time, ICU stay, hospital stay time, and hospital mortality in the reference centers of the state of São Paulo. Furthermore, there was a significant increase in the use of BIMA and radial artery grafts^[13]^. It is important to note that this registry used the Society of Thoracic Surgeons database and underwent internal and external quality audits to assess the completeness, consistency, and accuracy of the data. This project introduced the concept of rescue failure rate^[14]^. This quality metric explains that even hospitals with the same complication rate may present different rescue failure rates and, consequently, different mortality rates. Research has shown that many post-CABG deaths are not due to technical errors in the operating room, but to the inability of multidisciplinary teams to manage complications after CABG surgery^[15]^. These concepts broaden this discussion, highlighting that, in addition to a highly specialized cardiac surgeon, the results will depend on a multidisciplinary team acting as a care pathway. And it will be the cardiac surgeon specializing in CABG who will lead and align a team of specialists: anesthesiologists, perfusionists, intensivists, physiotherapists, nurses, and cardiologists who, following evidence-based protocols, will offer personalized care that is indispensable for the state of the art and the generation of value throughout the country, functioning as true CABG care pathways in centers of excellence.

## The State of the Art: Tailoring the Procedure through Dedicated Coronary Surgery Training

CABG is becoming a superspeciality due to its technical complexity, the need for precision, detailed anatomical knowledge, evaluation of imaging studies, management of complicated cases, interdisciplinary relationships, attention to the phases of care, and long-term results. The technique requires delicacy to preserve the endothelium, avoiding thrombosis, with special attention to the handling of grafts. The operation involves a repetitive choreography, where skill in performing anastomoses is crucial, especially under time constraints, with fine sutures and the use of a magnifying glass. In-depth knowledge of epicardial topography and anatomical variations is fundamental to identifying and preparing the coronary vessels^[16]^.

Emphasizing the necessity for focused specialization, we propose the establishment of teams dedicated exclusively to CABG, mirroring models seen in cancer or stroke centers. Such centers would uphold commitments to best practices, transparency in outcomes, and active participation in research initiatives. The formation of a national or international network of advanced centers could facilitate the adoption of contemporary techniques and bolster collaborative training programs.

**Fig. 1:**
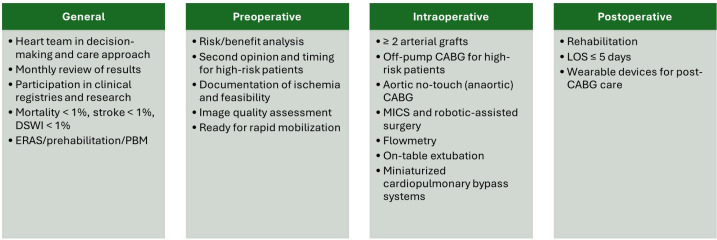
State of the art in the care of patients undergoing elective isolated coronary artery bypass grafting (CABG). DSWI=deep sternal wound infection; ERAS=enhanced recovery after surgery; LOS=length of stay; PBM=patient blood management

A core pillar of this superspeciality is the *state of the art* approach, which moves away from a one-size-fits-all surgery toward the personalization of the technique. Central to this is the meticulous selection of grafts; while the left internal mammary artery remains the cornerstone, the specialized use of BIMAs and radial grafts is essential for ensuring long-term graft patency and survival, particularly in younger patients. Furthermore, the mastery of OPCAB surgery represents a critical skill set in the superspecialist’s arsenal. By avoiding cardiopulmonary bypass, surgeons can significantly mitigate the systemic inflammatory response, reducing the risk of stroke and renal failure in high-risk subgroups. When this technical precision is married to minimally invasive and robotic platforms, the surgery reaches its highest expression of refinement. This synergy allows for a tailored strategy - whether it is a hybrid approach or a totally endoscopic procedure - ensuring that the surgical trauma is minimized while the revascularization remains complete and durable^[17,18]^ (Figure 1).

## The Technological Frontier: Democratizing Precision through Mini-Aggression

While advancements have been made in cardiac surgery specialization at major medical centers, coronary procedures are often treated as general practices. This approach contradicts evidence suggesting that dedicated training in CABG is critical for improving outcomes. The transition to a superspeciality is not an elitist movement but a technological upgrade for the entire surgical class. By focusing on minimal invasive, we provide surgeons with a toolkit to address the modern patient profile: complex anatomy, older, more fragile, female, and with higher expectations.

Minimally invasive cardiac surgery (MICS) utilizes small lateral thoracotomies. This approach demands a deeper understanding of thoracoscopic anatomy. Superspecialization provides the fellowship framework to master these skills safely. For the economically active population, this means returning to their livelihood in weeks rather than months, a crucial public health outcome.

Robot-assisted CABG is currently a reality, with a learning curve for improving surgical technique safely. Robotics offers 10× magnification and 7 degrees of freedom, allowing for totally endoscopic CABG^[19]^. This precision is essential for harvesting BIMA - the gold standard for long-term patency. Instead of being a proprietary technique, we propose *robotics centers*, where experienced surgeons guide general practitioners, facilitating learning and ensuring that technology serves the patients, not just the institution.

## Perfecting Surgical Journey with the Enhanced Recovery After Surgery Revolution and Patient Blood Management

Public policy must be patient-centered. The implementation of enhanced recovery after surgery (ERAS) protocols in CABG is a game-changer^[20]^. By standardizing preoperative nutrition, incorporating the concept of patient blood management (PBM), multimodal analgesia, and ultra-early mobilization, ERAS has been shown to reduce hospital stays by an average of two days in Latin American scenario^[21]^. Integrating ERAS and PBM into the SUS framework would not only improve the patient experience but also optimize resource allocation in a system already under strain^[22]^.

In a superspecialized service, surgery begins weeks before the incision with nutritional support, physiotherapy protocol, and psychological assessment to complement pre-rehabilitation, as well as the management of anemia. During the procedure, the focus shifts to opioid-sparing through multimodal analgesia and goal-directed fluid therapy. By using regional blocks (pectoralis/erector spinae plane blocks among others), the patient can be awake and breathing spontaneously minutes after surgery^[23]^.

Success is measured by the patient sitting up and drinking liquids within six to 12 hours postoperatively. This requires a culture shift that only a superspecialized department can sustain, transforming the patient from a *passive recipient* into an *active participant* in their recovery, aligning Brazilian practice with the best centers in the world.

## Science-Led Policy: Multicenter Randomized Trials

Public policies should be guided by data. Initiatives such as the BraSCORE, which aim to identify opportunities for improving care and implementing cost-effective strategies, need to be recognized for their importance^[24]^. Similarly, prospective, randomized multicenter studies like the FRAGILE trial seek to improve outcomes in frail patients, a condition that accompanies population aging and is increasingly prevalent^[25]^. The QUEEN study aims to improve outcomes in women through the selection of the best grafts in CABG surgery^[26]^. Evidence from studies focused on contemporary outcomes such as rapid recovery and quality of life for patients is increasingly necessary.

## Call to Action: A National Covenant for Coronary Excellence

We call upon the Brazilian Ministry of Health and surgical societies to initiate a national covenant based on:

1. Formal certification: Creating a pathway for *advanced coronary surgery* to empower surgeons.
2. Institutionalization of ERAS and PBM: To make perioperative optimization and the philosophy of least invasiveness the national standard for Brazil.
3. Regionalization: Creating a *hub-and-spoke* network for mentorship and MICS and robotic technology sharing.
4. Data transparency: Mandating participation in registries to reduce regional variability and promote accountability.

## Proposed Strategies for Recognition and Implementation

A structured approach with strong leadership, standardized protocols, and quality audits could propel isolated and elective CABG practices toward achieving operational mortality rates below 1%^[27]^. Initiatives to establish specialized training programs as well as fellowship options for new specialists are crucial.

CABG is a well-studied and frequently performed surgical procedure, characterized by its technical complexity and the demand for precision. It necessitates a comprehensive understanding of anatomy, adeptness in managing imaging assessments, and the ability to navigate challenging cases collaboratively across disciplines. Furthermore, postoperative care, assessment, and long-term outcomes are all crucial phases requiring concentrated attention. Specialized programs lead to enhanced expertise, quicker operations, increased arterial graft usage, fewer complications, and improved survival rates^[28]^. The transformation of CABG into a superspecialty is a commitment to ensuring that the *gold standard* of care is not a privilege of the few, but a right for every Brazilian citizen.

The CABG-specialized cardiac surgeon is the starting point for building coronary surgery teams, multidisciplinary teams composed of professionals specialized in the state-of-the-art care of patients undergoing myocardial revascularization surgery.

## Data Availability

The authors declare that the data supporting the findings of this study are available within the article.

## Artificial Intelligence Usage

The authors declare the use of Genspark AI 4.0 for the creation of figures and illustrations. The content generated by the artificial intelligence tool was reviewed and edited by the authors as necessary, and they take full responsibility for the final content to be published.
